# Total dietary fat and omega-3 fatty acids have modest effects on urinary sex hormones in postmenopausal women

**DOI:** 10.1186/1743-7075-10-36

**Published:** 2013-04-23

**Authors:** Lindsay R Young, Susan K Raatz, William Thomas, J Bruce Redmon, Mindy S Kurzer

**Affiliations:** 1 Department of Food Science and Nutrition, University of Minnesota, Minneapolis, MN, USA; 2 Department of Biostatistics, University of Minnesota, Minneapolis, MN, USA; 3 Department of Medicine, University of Minnesota, Minneapolis, MN, USA; 4 USDA Grand Forks Human Nutrition Research Center, 2420 2nd Ave N, Grand Forks, ND, 58203, USA

**Keywords:** Dietary fat, Omega-3 fatty acids, Low fat diet, Urinary estrogens, Postmenopausal women

## Abstract

**Background:**

Total fat and omega-3 fatty acids in the diet may affect breast cancer risk by altering estrogen metabolism. The purpose of this study was to elucidate the effects of differing total fat and omega-3 fatty acid content of diets on a panel of urinary estrogens and metabolites.

**Findings:**

A controlled, cross-over feeding trial was conducted in postmenopausal women using three test diets: high fat diet (HF; 40% energy from fat), low fat diet (LF; 20% of energy from fat) and low fat, high omega-3 diet (LFn3; 23% energy from fat; 3% omega-3 fatty acids) for 8 week periods. Urinary hormone concentrations for 16 women were compared among diets using a linear mixed model, and within diet comparisons were made using paired t-tests. Urinary excretion of estrone was greater after the LF and LFn3 compared to the HF (P = 0.004). Estrone excretion was increased from baseline within the LF only (P = 0.02). Total estrone + estradiol + estriol increased from baseline with LF (P = 0.02) and was greater than the other two diets at 8 weeks (P = 0.03). There were no effects on estrogen metabolites, including the 2-hydroxy estrone:16α-hydroxy estrone ratio.

**Conclusions:**

The results of this study indicate that urinary sex hormone metabolism was modestly altered in postmenopausal women by a low fat dietary intervention.

## Finding

### Introduction

High concentrations of estrogens in blood and urine are associated with increased risk of breast cancer in postmenopausal women [1], and one hypothesis is that this increased risk is mediated by sex hormone metabolism. Low fat diets resulted in significant reductions in circulating estrogens in some studies in postmenopausal women [2-4]. Dietary omega-3 fatty acids (n-3) may suppress tumorigenesis by inhibition of inflammatory eicosanoids, suppression of aromatase, and conversion of androgens to estrogens [5].

In addition to promoting growth via interaction with estrogen receptors, circulating estrogens are metabolized by hydroxylation reactions resulting in an array of metabolites with varying biological activity relative to the parent estrogens, with some having genotoxic activity [6]. Sixteen-α-hydroxy estrone (16αOH-E1) is a potent mitogen, tumor initiator, and tumor promoter *in vitro*[7]. 16α-hydroxylation of estrogens was enhanced in women with breast cancer relative to healthy controls [8,9]. A higher ratio of 2-hydroxy estrone (2OH-E1) to 16αOH-E1 (2:16αOH-E1 ratio) indicated decreased breast cancer risk in some studies [10,11]; however, other investigations do not support this association [12,13].

We have previously shown that a high fat diet elevates plasma estradiol [14]. The purpose of the present study was to determine the effects of diets with varying amounts of fat and n-3 on urinary estrogens and estrogen metabolites in healthy postmenopausal women. We hypothesized that compared to a high fat diet, diets low in fat or low in fat with high n-3 would decrease individual and total estrogens, 16αOH-E1, and the carcinogenic 4-hydroxy metabolites.

### Subjects and methods

#### Experimental protocol

Complete details of the study design and treatments are described elsewhere [14]. The effects of controlled test diets: a high fat diet (HF; 40% energy as fat, 15% energy as protein, and 45% energy as carbohydrate), a low fat diet (LF; 20% energy as fat, 15% energy as protein, and 65% energy as carbohydrate) and a low fat diet with n-3 fatty acids (LFn3; 23% energy as fat [3% n-3], 15% energy as protein, and 62% energy as carbohydrate) on urinary estrogen metabolism were compared in a randomized, cross-over trial. All nutrient analysis was performed by a registered dietitian (SKR) using the Nutritionist V nutrient analysis program (Axxya Systems, Stafford, TX). Subjects consumed each diet in random order for 8 weeks with a 6–12 week washout between treatments. Reported dietary compliance was >99% for energy and >99.5% for omega-3 fatty acids [14].

The study was carried out from 2004–2008. The study was approved by the U.S. Army Medical Research and Materiel Command’s Human Subjects Research Review Board and the University of Minnesota Committee for the Use of Human Subjects in Research. Written informed consent was obtained from all subjects. The trial was retrospectively registered at http://www.clinicaltrials.gov as NCT01824498.

#### Subjects

Subject characteristics and recruitment are discussed elsewhere [14]. Participants were healthy postmenopausal women; age 45 – 70 years; had a body mass index of 19 – 32 kg/m^2^; had not lost or gained more than 5 pounds in the last 6 months; and had not used fish oil supplements or hormone replacement therapy for 2 months prior to the trial. Subjects were excluded if they had any active disease process, used prescription medications, or had both ovaries removed. None of the participants had prior a prior breast cancer diagnosis.

**Figure 1:**
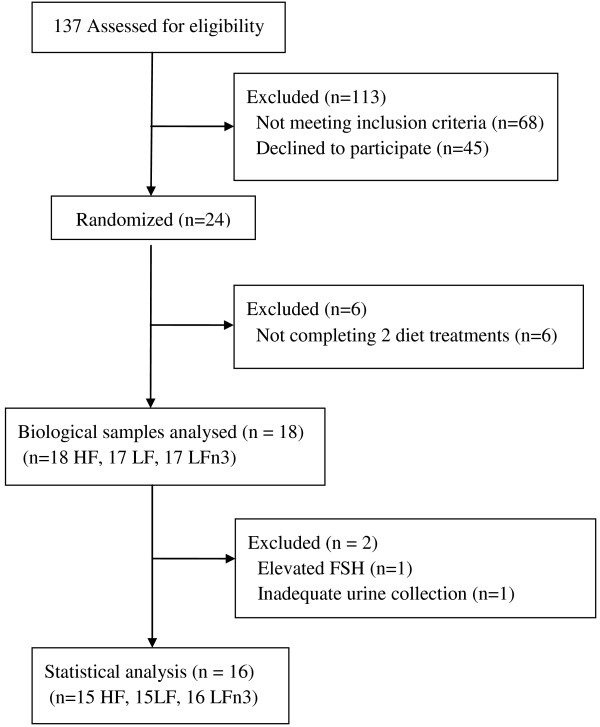
Flow diagram of participants from screening to data analysis.

Seventeen subjects completed all three dietary treatments and one subject completed two treatments. One subject was excluded from the analysis because her follicle stimulating hormone concentration was intermittently in the premenopausal range. Another subject was excluded due to improperly collected urine specimens. One subject was missing urine samples for HF. In total, 15 subjects completed HF, 15 subjects completed LF, and 16 subjects completed LFn3 and are included in the final analysis. Figure 1 illustrates the flow of participants through the trial.

#### Urine collection

Subjects collected two consecutive 24-hour urine specimens at home on weeks 0 and 8 of each treatment period. Urine was pooled and sodium azide was added to 0.1%. Aliquots were immediately stored at −20°C until analysis.

#### Plasma phospholipid fatty acids

Plasma phospholipid fatty acids (PLFA) were analyzed by gas chromatography (Lipid Technologies, Austin, MN) as previously reported (14). Analysis was done to determine the effects of dietary treatments on PLFA content (μg/ml) of linoleic acid (LA, 18:2n6), α-linolenic acid (ALA, 18:3n3), arachidonic acid (AA, 20:4n6), eicosapentaenoic acid (EPA, 20:5n3) and docosahexaenoic acid (DHA, 22:6n3), total n3, total n6 and the n3/n6 ratio.

#### Estrogen metabolite assay by LC/MS-MS

LC-MS methods were used to determine urinary estrogens and estrogen metabolites [15,16]. Unless otherwise noted, all laboratory chemicals were obtained from Fisher Scientific (Pittsburg, PA). 10 μL of deuterated-estrogen metabolite internal standard solution (ISS; C/D/N Isotopes, Inc., Pointe-Claire, Quebec, Canada) containing representative analytes of each class of compounds were added to duplicate 1.0 ml urine aliquots, followed by 1.0 mL of freshly prepared enzymatic hydrolysis buffer containing 2 mg of L-ascorbic acid, 5 μL of β-glucuronidase/sulfatase (Type H-2; Sigma-Aldrich, St Louis, MO), and 1.0 mL of 0.15 M sodium acetate buffer (pH 4.1) and incubated overnight at 37°C.

Samples were extracted with ethyl ether and evaporated to dryness at 40°C under nitrogen. 25 μL of 0.1 M sodium bicarbonate buffer with 0.1% ascorbic acid (pH at 9.0) and 25 μL of dansyl chloride solution (3 mg/mL in acetone; Lancaster Synthesis, Inc., Pelham, NH) were added to each dried sample. Each sample was heated at 65°C for 6 minutes to form estrogen metabolite and deuterated estrogen metabolite dansyl derivatives. Calibration standards (Steraloids, Inc., Newport, RI) and quality control samples were similarly hydrolyzed, extracted, and derivatized.

Chromatographic separation was performed on a 100 × 0.5 mm (i.d.) Zorbax SB-C18 column 1.8μm particle size (Agilent Technologies, Santa Clara, CA). The mobile phase consisted of two eluents, solvent A (50 mL/L acetonitrile + 950 mL/L H2O containing 1 mL/L formic acid) and solvent B (475 mL/L acetonitrile + 475 mL/L methanol + 50 mL/L H2O containing 1 mL/L formic acid). LC/MS-MS analysis was performed on a Thermo Electron Quantum Discovery Max Triple Quadrupole Instrument. Quantitative analysis was performed using Thermo Electron Xcalibur proprietary software. All samples from a given subject were batch analyzed. Intra-batch coefficients of variation varied from 5.1 – 12.2%. Inter-batch coefficients of variation for quality control samples varied from 6.3 – 27.9% among hormones.

#### Statistical analysis

Data for urinary sex hormone metabolites were not normally distributed and are reported as geometric means and 95% confidence intervals. SAS Proc Mixed (SAS version 9.2, SAS Institute Inc., Cary, NC; 2002–2008) was used to fit a mixed effects linear model for each outcome with a random effect for subject to account for multiple correlated measurements within each subject. These effect models handled isolated missing values and were used to assess period and carryover effects. Least squares means generated from the mixed model were compared between treatments. Paired t-tests were applied to within diet differences. A P-value of < 0.05 was considered statistically significant.

### Results

Study participants were slightly overweight (mean BMI 27 kg/m^2^, Table 1) mostly white (88%) middle-aged women (mean age 58 ± 6 years).

**Table 1:** **Characteristics of study subjects at baseline**^**a**^

**Characteristic**	**Value**
Age (years)	58 ± 6
Body weight (kg)	74 ± 11
Height (cm)	165 ± 5
BMI (kg/m^2^)	27 ± 4
Systolic blood pressure (mmHg)	118 ± 15
Diastolic blood pressure (mmHg)	71 ± 8
Follicle stimulating hormone (mIU/mL)^b^	73 ± 22
Ethnicity (n (%))	
White	14 (88)
Hispanic	1 (6)
African-American	1 (6)

^a^All values are means ± standard deviations except ethnic groups, which are n (%); n = 16.

^b^Baseline follicle stimulating hormone values for all women ≤ 55 years old; n = 11.

#### Plasma phospholipid fatty acids

The results for n-3 and n-6 in PLFA are presented in Table 2. The LFn3 diet, which was low fat with supplemental n-3 promoted enhanced inclusion of total n-3, ALA, EPA and DHA into PLFA compared to the HF and LF diets (all p < 0.0001). The increases in n-3 were accompanied by a reduction in total n-6, LA, and ARA. In comparison to HF, LF promoted increased ARA and DHA (p=.02, p=.03, respectively).

**Table 2:** **Baseline, 8 weeks, and change from baseline to 8 weeks concentrations for n3 and n6 plasma phospholipid fatty acids in μg/ml**^**1**^

	**High fat**	**Low fat**	**Low fat + n-3**	
	**n = 17**	**n = 16**	**n = 17**	**p-value**^**2**^
18:2n6				
Baseline	280.69 ± 14.57	284.66 ± 14.76	293.95 ± 14.59	0.41
8 weeks	285.49 ± 12.54^a^	269.21 ± 12.86^a^	233.08 ± 12.59^b^*	0.0006
Δ 8 weeks	4.81 ± 10.90^a^	−16.08 ± 11.29^a^	−60.85 ± 10.95^b^	0.0001
p-value^3^	0.66	0.16	< 0.0001	
18:3n3				
Baseline	4.00 ± 0.86	4.51 ± 0.88	3.22 ± 0.86	0.34
8 weeks	2.57 ± 0.45^a^	3.12 ± 0.47^a^	6.85 ± 0.45^b^*	< 0.0001
Δ 8 weeks	−1.43 ± 0.79^a^	−1.30 ± 0.82^a^	3.62 ± 0.79^b^	< 0.0001
p-value^3^	0.08	0.12	< 0.0001	
20:4n6				
Baseline	120.62 ± 7.83	121.51 ± 7.94	126.04 ± 7.84	0.59
8 weeks	107.89 ± 7.14^a^*	135.62 ± 7.34^b^*	100.12 ± 7.17^a^*	0.0001
Δ 8 weeks	−12.72 ± 5.13^a^	13.47 ± 5.33^b^	−25.90 ± 5.16^a^	< 0.0001
p-value^3^	0.02	0.02	< 0.0001	
20:5n-3				
Baseline	11.43 ± 1.77	11.80 ± 1.82	9.69 ± 1.78	0.49
8 weeks	6.89 ± 1.80^a^*	9.99 ± 1.87^a^	29.86 ± 1.81^b^*	< 0.0001
Δ 8 weeks	−4.54 ± 1.94^a^	−1.91 ± 2.02^a^	20.17 ± 1.96^b^	< 0.0001
p-value^3^	0.02	0.35	< 0.0001	
22:5n-3				
Baseline	9.94 ± 0.78	10.56 ± 0.79	10.72 ± 0.78	0.49
8 weeks	8.41 ± 0.88^a^*	11.04 ± 0.91^b^	13.45 ± 0.89^c^*	< 0.0001
Δ 8 weeks	−1.52 ± 0.69^a^	0.36 ± 0.72^a^	2.73 ± 0.70^b^	< 0.0001
p-value^3^	0.03	0.62	0.0003	
22:6n-3				
Baseline	30.18 ± 2.82	28.90 ± 2.89	29.68 ± 2.83	0.89
8 weeks	24.06 ± 3.77^a^*	35.03 ± 3.90^b^*	55.00 ± 3.78^c^*	< 0.0001
Δ 8 weeks	−6.12 ± 2.56^a^	5.95 ± 2.65^b^	25.32 ± 2.57^c^	< 0.0001
p-value^3^	0.02	0.03	< 0.0001	
Total n-3				
Baseline	59.71 ± 5.33	60.07 ± 5.42	56.75 ± 5.34	0.68
8 weeks	44.50 ± 6.37^a^*	62.86 ± 6.60^b^	110.10 ± 6.41^c^*	< 0.0001
Δ 8 weeks	−15.21 ± 4.95^a^	2.55 ± 5.14^b^	54.36 ± 4.98^c^	< 0.0001
p-value^3^	0.004	0.62	< 0.0001	
Total n-6				
Baseline	452.87 ± 21.30	456.78 ± 21.57	468.55 ± 21.34	0.53
8 weeks	432.99 ± 18.17^a^	458.41 ± 18.74^a^	370.63 ± 18.25^b^*	0.0005
Δ 8 weeks	−19.85 ± 14.82^a^	−0.58 ±15.34^a^	−97.85 ± 14.89^b^	< 0.0001
p-value^3^	0.19	0.97	< 0.0001	

^1^All values are LS means ± standard error.

^2^p-value for effect of treatment across the three diets evaluated using a general linear mixed model. Values with differing letters as a superscript are significantly different at p < 0.05.

^3^p-value for paired t-test comparing baseline and 8 weeks means within each diet.

^*^8 week mean is significantly different from baseline mean.

#### Urinary sex hormones

Urinary estrone (E1) excretion was significantly greater after LF and LFn3 compared to HF (P = 0.004, Table 3), although E1 excretion was significantly increased from baseline with LF only (P = 0.02). Excretion of the sum of E1, estradiol (E2) and estriol (E3) was significantly greater with LF than HF at 8 weeks (P = 0.03) and E1+ E2 + E3 increased significantly from baseline to 8 weeks with LF (P = 0.02). Urinary excretion of 2OH-E1, 2-methoxy estrone (2-ME1), 2-methoxy estradiol (2-ME2), 4-hydroxy estrone (4OH-E1), 16αOH-E1, 2:16αOH-E1 ratio and total estrogens were not significantly different among test diets at 8 weeks. Urinary 2-hydroxy estradiol (2OH-E2), 4-hydroxy estradiol (4OH-E2), 4-methoxy estrone (4-ME1), and 4-methoxy estradiol (4-ME2) were undetectable in urine.

**Table 3:** **Baseline and 8 week values for urinary estrogens and metabolites for the HF, LF and LFn3**^**a**^

**Analyte**	**HF**	**LF**	**LFn3**	**P-value**^**d**^
	**n = 15**^**b**^	**n = 15**^**c**^	**n = 16**	
Estrone (E1) (nmol/day)				
Baseline	6.7 (4.8, 9.5)	7.2 (5.1, 10.1)	8.1 (5.8, 11.5)	0.36
8 weeks	6.3 (4.4, 9.1) ^§^	9.9 (6.9, 14.2) ^†^**	8.5 (5.9, 12.1) ^†^	0.004
P-value^e^	0.39	0.02	0.92	
Estradiol (E2) (nmol/day)				
Baseline	2.0 (1.0, 3.8)	2.2 (1.2, 4.3)	1.4 (0.7, 2.6)	0.36
8 weeks	1.0 (0.5, 2.1)	2.1 (1.0, 4.4)	1.7 (0.8, 3.4)	0.12
P-value^e^	0.37	0.47	0.83	
Estriol (E3) (nmol/day)				
Baseline	7.2 (3.1, 16.8)	8.5 (3.6, 19.9)	10.8 (4.7, 25.0)	0.62
8 weeks	11.8 (6.3, 22.0)	14.3 (7.7, 26.7)	11.7 (6.3, 21.5)	0.79
P-value^e^	0.84	0.12	0.64	
E1 + E2 + E3 (nmol/day)				
Baseline	22.9 (15.3, 34.4)	24.8 (16.6, 37.1)	23.0 (15.4, 34.2)	0.77
8 weeks	21.5 (14.3, 32.2)^§^	35.7 (23.9, 53.4)^†^**	26.8 (18.1, 39.8)^§†^	0.03
P-value^e^	0.51	0.02	0.60	
2-hydroxyestrone (2OH-E1) (nmol/day)				
Baseline	22.7 (16.5, 31.2)^§†^	25.0 (18.2, 34.4)^§^	18.5 (13.5, 25.3)^†^	0.02
8 weeks^f^	17.6 (13.7, 22.7)	20.4 (15.8, 26.3)	24.1 (18.8, 30.9)	0.23
P-value^e^	0.19	0.81	0.29	
2-methoxyestrone (2-ME1)(nmol/day)				
Baseline	1.6 (0.7, 3.8)	3.4 (1.5, 7.8)	3.1 (1.4, 7.1)	0.21
8 weeks	1.4 (0.7, 3.1)	2.1 (0.9, 4.5)	3.3 (1.5, 7.1)	0.23
P-value^e^	0.98	0.48	0.91	
2-methoxyestradiol (2-ME2) (nmol/day)				
Baseline	1.3 (0.6, 3.3)	3.9 (1.6, 9.4)	1.7 (0.7, 3.9)	0.09
8 weeks	0.9 (0.4, 2.1)	1.0 (0.4, 2.3)	1.7 (0.8, 4.0)	0.39
P-value^e^	0.84	0.08	0.57	
4-hydroxyestrone (4OH-E1) (nmol/day)				
Baseline	0.78 (0.51, 1.21)	1.08 (0.70, 1.68)	1.17 (0.76, 1.80)	0.21
8 weeks	0.92 (0.66, 1.27)	1.05 (0.76, 1.45)	1.28 (0.93, 1.75)	0.25
P-value^e^	0.64	0.93	0.50	
16α-hydroxyestrone (16αOH-E1) (nmol/day)				
Baseline	2.03 (1.10, 3.75)	2.39 (1.29, 4.44)	1.70 (0.93, 3.11)	0.62
8 weeks	1.50 (0.82, 2.74)	2.34 (1.27, 4.29)	2.03 (1.12, 3.65)	0.52
P-value^e^	0.47	0.22	0.32	
Total Estrogens (nmol/day)				
Baseline	73.7 (52.7, 100.1)	83.7 (60.9, 115.0)	72.8 (53.1, 99.6)	0.38
8 weeks	65.5 (45.8, 93.8)	86.4 (60.7, 123.0)	80.6 (57.0, 113.9)	0.27
P-value^e^	0.78	0.54	0.40	
2:16αOH-E1 ratio (nmol/day)				
Baseline	11.3 (5.4, 23.6)	10.5 (5.0, 21.9)	10.8 (5.3, 22.4)	0.98
8 weeks	11.9 (5.7, 25.2)	10.0 (4.7, 21.3)	10.4 (5.0, 21.7)	0.90
P-value^e^	0.90	0.12	0.72	

HF: high fat diet; LF: low fat diet; LFn3: low fat + omega-3 fatty acids diet; E1: estrone;

2:16αOH-E1 ratio: 2-hydroxy estrone:16α-hydroxy estrone ratio.

^a^All values are geometric means (95% confidence interval).

^b^One subject was missing urines for HF diet, thus n = 15 for HF.

^c^One subject was missing urines for LF diet, thus n = 15 for LF.

^d^P-value for effect of treatment across the three diets; values with differing symbols (§,†) as a superscript are significantly different.

^e^P-value for paired t-test comparing baseline and 8 weeks means within each diet.

**8 weeks mean is significantly different from baseline mean.

^f^8 weeks means were adjusted for baseline 2OH-E1.

### Discussion

Estrone excretion was significantly greater with LF and LFn3 compared to HF, although there was little overall change in urinary hormone metabolites. The sum of all estrogens and metabolites assayed in this study (Total Estrogens) did not differ among diets at 8 weeks. We previously reported the effects of the test diets on plasma sex hormones [14]. In blood, E2 concentration increased significantly with HF (P = 0.03) and decreased slightly, but non-significantly with LF (P = 0.10) [14]. Therefore, we anticipated changes in sex hormone metabolism measured in urine. We saw no change in urinary sex hormone metabolite excretion even though changes in the parent hormones E1 and E2 were observed. Limiting factors include the high variability (wide confidence intervals) of urinary hormones and our small sample size.

We anticipated that LF and LFn3 would reduce excretion of estrogens and metabolites in accordance with the hypothesis that a low fat diet alters profiles of sex hormones in a direction of reduced breast cancer risk. Increased excretion of urinary estrogens (defined as E1 + E2 + E3 or E1 + E2) was associated with increased risk of breast cancer in another study of postmenopausal women [1]. Decreased excretion of estrogens was observed in postmenopausal women from Asian nations with relatively low breast cancer incidence and concomitantly low intake of dietary fat [17-19].

In addition to total dietary fat, n-3 may play a role in breast cancer prevention. Greenland Eskimos, with high n-3 intake [20], have historically had a low incidence of breast cancer [21,22]. However, this risk has increased as Westernized and nontraditional foods have become more prevalent in their diets [23]. Few studies have investigated the relationship of n-3 and urinary sex hormone metabolism in humans. Urinary 16αOH-E1 was reduced in the n-3 fatty acid supplement arm in a pilot trial of high-risk women [24]. In a study by Wu et al. [25] postmenopausal vegetarian women consuming 2.14 g/day of n-3 docosahexaenoic acid had no significant change in urinary 2:16αOH-E1 ratio following intervention. In our study, 2:16αOH-E1 ratio was not altered following 8 weeks of dietary intervention.

### Conclusion

In conclusion, urinary sex hormone metabolism was modestly altered in healthy postmenopausal women by LF alone or with additional n-3.

## Abbreviations

2OH-E1: 2-hydroxy estrone; 2OH-E2: 2-hydroxy estradiol; 2-ME1: 2-methoxy-estrone; 2-ME2: 2-methoxy estradiol; 2:16αOH-E1 ratio: 2-hydroxy estrone:16α-hydroxy estrone ratio; 4OH-E1: 4-hyrdroxy estrone; 4OH-E2: 4-hydroxy estradiol; 4-ME1: 4-methoxy estrone; 4-ME2: 4-methoxy estradiol; 16αOH-E1: 16-alpha-hydroxy estrone; E1: Estrone; E2: Estradiol; E3: Estriol; HF: High fat diet; LF: Low fat diet; LFn3: Low fat + omega-3 fatty acids diet; n3: Omega-3; n6: Omega 6; ALA: α-linolenic acid; EPA: Eicosapentaenoic acid; DHA: Docosahexaenoic acid; LA: Linolenic acid; ARA: Arachidonic acid.

## Competing interests

The authors declare that they have no competing interests.

## Authors’ contributions

The authors’ responsibilities were as follows - SKR and MSK designed the research; SKR, LRY and JBR conducted the research; WT, LRY and SKR analyzed the data; LRY, SKR and MSK wrote the manuscript; all authors had responsibility for final content. All authors read and approved the final manuscript.
